# Political identity, algorithmic news exposure, and trust in digital media: the sequential mediating associations of motivated reasoning and misinformation susceptibility

**DOI:** 10.3389/fpsyg.2026.1900098

**Published:** 2026-08-26

**Authors:** Sixuan Liu

**Affiliations:** The Australian National University, Canberra, ACT, Australia

**Keywords:** algorithmic news exposure, digital news, misinformation susceptibility, motivated reasoning, political communication, political identity, structural equation modeling, trust in digital media

## Abstract

**Background:**

Digital media have become a major environment in which citizens encounter political news, evaluate public affairs, and form judgments about information credibility. In algorithmically mediated news environments, trust in digital media may depend not only on platform exposure but also on how users process identity-relevant political information.

**Objective:**

This study examined how political identity and algorithmic news exposure were associated with trust in digital media through motivated reasoning and misinformation susceptibility.

**Methods:**

A cross-sectional survey was conducted among 420 adult digital news users recruited through an online survey panel. Political identity, algorithmic news exposure, motivated reasoning, and trust in digital media were measured using multi-item scales. Misinformation susceptibility was assessed using a composite score derived from self-report items and a headline judgment task containing accurate and misleading political or public-affairs headlines. Data were analyzed using partial least squares structural equation modeling with 5,000 bootstrap samples. PROCESS Model 6 was used as a robustness check. Age, gender, education, political interest, digital news use frequency, and media literacy were included as control variables.

**Results:**

Political identity was positively associated with motivated reasoning (β = 0.359, *p* < 0.001), and algorithmic news exposure was also positively associated with motivated reasoning (β = 0.115, *p* = 0.010). Motivated reasoning was positively associated with misinformation susceptibility (β = 0.335, *p* < 0.001). Misinformation susceptibility was negatively associated with trust in digital media (β = −0.283, *p* < 0.001). The sequential indirect association from political identity to trust in digital media through motivated reasoning and misinformation susceptibility was statistically supported (β = −0.034, 95% CI [−0.053, −0.019]). The corresponding sequential indirect association from algorithmic news exposure to trust in digital media was also supported, although weaker in magnitude (β = −0.011, 95% CI [−0.021, −0.003]). The direct associations from political identity and algorithmic news exposure to trust in digital media were not statistically supported after the mediators and controls were included.

**Conclusion:**

The findings suggest that trust in digital media is associated with both identity-based reasoning and algorithmically structured news exposure, but primarily through motivated reasoning and misinformation susceptibility. The study suggests that trust in digital media is associated with cognitive and informational processes, such as motivated reasoning and misinformation susceptibility, rather than functioning solely as a direct correlate of platform use or political identity.

## 1 Introduction

Digital media have become a central environment through which citizens encounter political information, evaluate public affairs, and form judgments about the credibility of news. Unlike earlier news systems, where editorial institutions played a more visible gatekeeping role, contemporary digital platforms organize political information through personalized feeds, recommender systems, social sharing, trending lists, and algorithmic ranking. This shift has co-occurred with a news environment in which political exposure is shaped not only by users' active choices but also by platform infrastructures that determine which stories become visible, repeated, and socially amplified. Social media have been shown to play a significant role in the circulation of political misinformation during election periods (Allcott and Gentzkow, 2017).

The problem is not limited to the presence of false information online. Digital political communication also changes the conditions under which citizens process news. Political information is often encountered in emotionally charged, identity-relevant, and socially visible contexts. In such settings, citizens may not evaluate news only according to accuracy or evidentiary quality. Instead, prior beliefs, partisan attachments, ideological commitments, and group identity can influence whether information is accepted, rejected, or reinterpreted. Empirical evidence on Facebook sharing behavior indicates that fake news dissemination is unevenly distributed across users and is associated with political and demographic differences (Guess et al., 2019).

Political identity is therefore a key starting point for understanding trust in digital media. Political identity refers to the degree to which individuals define themselves through political affiliation, ideological orientation, or attachment to a political group. When political identity becomes salient, news evaluation may become tied to group belonging and self-concept. Information that confirms one's political worldview may be treated as more credible, while information that challenges the in-group may be discounted or perceived as biased. The identity-based model of political belief explains that social identity goals can shape information processing and may lead individuals to align beliefs with group commitments rather than factual accuracy (Van Bavel et al., 2024).

At the same time, algorithmic news exposure has changed how political identity is activated and reinforced. Algorithmic news exposure refers to the extent to which users encounter news through platform-based recommendation systems, personalized feeds, automated ranking, and engagement-driven content curation. Recommendation systems may repeatedly expose users to content that matches their prior interests, political preferences, emotional reactions, or engagement history. Evidence from YouTube shows that recommendation algorithms can significantly increase both news recommendations and actual news consumption (Yu et al., 2024). This means that algorithmic exposure should not be treated as a neutral technical background. It is part of the information environment in which users encounter, interpret, and evaluate political news.

A central psychological mechanism in this process is motivated reasoning. Motivated reasoning is defined as directional goal-driven cognition that prioritizes identity-consistent conclusions, often at the expense of normative inferential standards (Kunda, 1990). While Taber and Lodge describe this as “motivated skepticism”—a distinct pattern of harsher scrutiny toward counter-attitudinal information—our operationalization of motivated reasoning adopts a broader scope that encompasses both cognitive biases (selective acceptance) and identity-protective affective responses (Taber and Lodge, 2006). We recognize that items measuring a tendency to “question news that challenges political views” may overlap with epistemic skepticism; however, in the context of political identity, such scrutiny is theoretically interpreted as a defense mechanism rather than mere neutral inquiry. In political contexts, motivated reasoning can lead citizens to apply different standards to congenial and uncongenial information. Citizens may accept identity-consistent claims with limited scrutiny while demanding stronger evidence from identity-threatening claims. Political polarization research shows that group commitments and ideological attachments are closely connected with selective exposure, biased evaluation, and resistance to counter-attitudinal evidence (Jost et al., 2022). In digital media environments, these tendencies may become more consequential because political content is frequently embedded in personalized feeds, peer interaction, and emotionally engaging platform signals.

Motivated reasoning is also closely related to misinformation susceptibility. Misinformation susceptibility refers to individuals' tendency to believe, accept, or fail to distinguish false or misleading information from accurate information. Susceptibility to partisan fake news has been found to be better explained by insufficient reasoning than by partisan bias alone (Pennycook and Rand, 2019). This finding is important because it suggests that misinformation vulnerability is not simply a matter of political ideology. It may emerge when users fail to engage in careful evaluation or when identity-consistent content is processed with reduced skepticism. In political news environments, misinformation may therefore become especially persuasive when it fits what users already want to believe.

The final outcome of interest in this study is trust in digital media. Trust in digital media refers to users' confidence in the credibility, reliability, and integrity of digital news sources, platform-mediated information, and online news environments. Trust is increasingly fragile because users must evaluate not only journalistic sources, but also platform signals, algorithmic visibility, social endorsements, and the possibility of misinformation. Recent research highlights that digital media trust is heavily conditioned by psychosocial evaluations and demographic factors, including active user support for platform-based misinformation restrictions (Chung and Barry, 2026). This underscores the critical importance of accounting for user-level demographic baselines and proactive psychosocial evaluations when modeling these trust mechanics. Exposure to fake news has been linked to lower trust in mainstream media in longitudinal survey evidence (Ognyanova et al., 2020). In this sense, trust in digital media is not only a direct evaluation of media quality. It may also be an outcome of how users encounter political news, process identity-relevant information, and respond to misleading content.

Although prior studies have examined political identity, algorithmic curation, motivated reasoning, misinformation, and media trust, these research streams remain insufficiently integrated. Existing studies often treat political identity as an individual-level political factor, algorithmic exposure as a platform-level condition, misinformation susceptibility as a cognitive vulnerability, and media trust as a separate attitudinal outcome. This separation makes it difficult to explain how identity-based cognition and algorithmically structured exposure jointly relate to trust in digital media. Recent work on digital media systems argues that social drivers and algorithmic mechanisms should be examined together because user behavior and platform systems form mutually reinforcing feedback loops (Metzler and Garcia, 2024). Building on this perspective, the present study examines whether motivated reasoning and misinformation susceptibility explain how political identity and algorithmic news exposure are associated with trust in digital media.

This study responds to this gap in three ways. First, it brings political identity and algorithmic news exposure into the same empirical model, allowing the study to examine both identity-based and platform-structured correlates of digital media trust. Second, it specifies a sequential psychological mechanism by positioning motivated reasoning and misinformation susceptibility as mediating processes rather than treating misinformation vulnerability as an isolated outcome. Third, it extends media trust research by showing that trust in digital media may be associated with how users process political information before they evaluate the credibility of digital media environments. By testing this model with survey data, the study provides an empirically grounded account of how political identity, algorithmic exposure, motivated cognition, and misinformation susceptibility are connected to trust in digital media.

## 2 Literature review and hypothesis development

### 2.1 Political identity and motivated reasoning

Political identity provides individuals with a framework for interpreting political information. When people strongly identify with a political group or ideological position, political news may be evaluated not only as information but also as a signal of group status, moral alignment, or identity threat. Under these conditions, information processing becomes more likely to serve directional goals. Individuals may search for, remember, and evaluate evidence in ways that protect their prior commitments. The classic theory of motivated reasoning argues that motivation can bias the cognitive strategies individuals use to access, construct, and evaluate beliefs (Kunda, 1990). This theoretical logic suggests that stronger political identity should be associated with stronger motivated reasoning in the evaluation of digital political news.

*H1. Political identity is positively associated with motivated reasoning*.

### 2.2 Algorithmic news exposure and motivated reasoning

Algorithmic news exposure may also be positively associated with motivated reasoning because personalized feeds and recommendation systems can repeatedly present users with politically familiar, emotionally engaging, or identity-congruent content. Unlike direct news-seeking, algorithmic exposure is partly shaped by platform systems that infer preferences from prior behavior and engagement. This can create an information environment in which users encounter content that appears personally relevant and socially validated. A large-scale field experiment on social media news exposure showed that attempts to increase exposure to verified and ideologically balanced news were linked to only small and context-dependent changes in users' news engagement (Askari et al., 2024). This more cautious evidence suggests that algorithmically structured exposure can shape the conditions of news processing, although its behavioral effects may vary across users. When users frequently encounter news through personalized systems, they may become more likely to rely on identity-consistent interpretation rather than neutral evaluation.

*H2. Algorithmic news exposure is positively associated with motivated reasoning*.

### 2.3 Motivated reasoning and misinformation susceptibility

Motivated reasoning can increase misinformation susceptibility by weakening accuracy-oriented evaluation. In political contexts, users may treat congenial claims as more plausible and uncongenial claims as more questionable, even when both require careful verification. Research on motivated skepticism shows that citizens evaluate political arguments asymmetrically by accepting attitude-consistent arguments more readily and scrutinizing attitude-inconsistent arguments more harshly (Taber and Lodge, 2006). This creates a pathway through which false or misleading information can become persuasive when it supports a user's prior beliefs or political identity. Therefore, motivated reasoning is expected to be positively associated with vulnerability to misinformation.

*H3. Motivated reasoning is positively associated with misinformation susceptibility*.

### 2.4 Misinformation susceptibility and trust in digital media

Misinformation susceptibility may be negatively associated with trust in digital media because users who struggle to distinguish reliable from misleading information are more likely to experience uncertainty, confusion, and skepticism toward the digital news environment. Trust in digital media depends on users' confidence that online information systems can provide credible and reliable news. When users become more vulnerable to misinformation, this confidence may decline. A systematic meta-analysis of online misinformation susceptibility shows that misinformation judgments are associated with demographic and psychological factors and involve differences in both discrimination ability and response bias (Sultan et al., 2024). These findings suggest that susceptibility is not a trivial measurement issue but a meaningful individual difference that can affect how users evaluate the reliability of digital information environments.

*H4. Misinformation susceptibility is negatively associated with trust in digital media*.

### 2.5 The mediating role of motivated reasoning

Political identity may not be directly associated with misinformation susceptibility in every case. Rather, its influence is likely to operate through motivated reasoning. Strong political identity can make certain interpretations feel more acceptable, but motivated reasoning is the cognitive process through which identity is translated into selective evaluation. Individuals with strong political identities may become more susceptible to misinformation when they process political news in a directionally motivated way. The partisan brain framework explains that partisan identity can shape cognitive processes and help account for why individuals may place group loyalty above factual accuracy in political judgment (Van Bavel and Pereira, 2018). This supports the expectation that motivated reasoning mediates the relationship between political identity and misinformation susceptibility.

*H5. Motivated reasoning mediates the association between political identity and misinformation susceptibility*.

### 2.6 The mediating role of misinformation susceptibility

Motivated reasoning may also affect trust in digital media through misinformation susceptibility. When users evaluate news according to identity consistency rather than evidentiary quality, they may become more likely to accept misleading content or fail to identify false claims. Over time, this vulnerability may undermine confidence in digital news environments. Research on the psychological drivers of misinformation belief shows that misinformation endorsement is associated with cognitive, social, and affective factors and that corrected misinformation may continue to influence reasoning (Ecker et al., 2022). This indicates that misinformation susceptibility can have consequences beyond isolated belief errors. It can shape how users understand the reliability of the broader information environment.

*H6. Misinformation susceptibility mediates the association between motivated reasoning and trust in digital media*.

### 2.7 Sequential mediation from political identity and algorithmic news exposure to trust in digital media

The preceding arguments suggest a sequential mediation process. Political identity and algorithmic news exposure are expected to function as two antecedents of motivated reasoning. Motivated reasoning is expected to be positively associated with misinformation susceptibility. Misinformation susceptibility is then expected to be negatively associated with trust in digital media. This model of indirect associations reflects the idea that digital media trust is shaped not only by direct evaluations of news sources or platforms, but also by the psychological process through which users encounter and judge political information. Experimental evidence on social media recommendations shows that recommendations from opinion leaders can increase media trust and subsequent information-seeking intentions (Turcotte et al., 2015). This supports the broader assumption that digital media trust is sensitive to the way news is encountered, socially signaled, and interpreted.

*H7a. Motivated reasoning and misinformation susceptibility sequentially mediate the association between political identity and trust in digital media*.
*H7b. Motivated reasoning and misinformation susceptibility sequentially mediate the association between algorithmic news exposure and trust in digital media*.

### 2.8 Conceptual framework

Based on the above hypotheses, this study proposes a conceptual model in which political identity and algorithmic news exposure serve as initial predictor variables, motivated reasoning and misinformation susceptibility serve as sequential mediators, and trust in digital media serves as the outcome variable. The model assumes that stronger political identity and higher algorithmic news exposure are positively associated with motivated reasoning, which in turn is positively linked to misinformation susceptibility and negatively associated with trust in digital media. The study also controls for age, gender, education, political interest, digital news use frequency, and media literacy because these factors may influence political news processing and misinformation judgment. Prior research on online news consumption has shown that trust in news media is associated with source preferences and online participation behavior (Fletcher and Park, 2017). Therefore, including media-use-related controls can help reduce potential confounding when examining trust-related outcomes.

## 3 Method

### 3.1 Research design

This study adopted a cross-sectional quantitative survey design to examine the associations among political identity, algorithmic news exposure, motivated reasoning, misinformation susceptibility, and trust in digital media. The design was suitable because the study focused on individual-level perceptions, digital news exposure patterns, cognitive tendencies, and accuracy judgments in online political information environments. The empirical model included two antecedent variables, namely political identity and algorithmic news exposure; two sequential mediators, namely motivated reasoning and misinformation susceptibility; and one outcome variable, namely trust in digital media. Age, gender, education, political interest, digital news use frequency, and media literacy were included as control variables because these factors may shape political news use, misinformation judgment, and trust-related evaluations.

**Figure 1:**
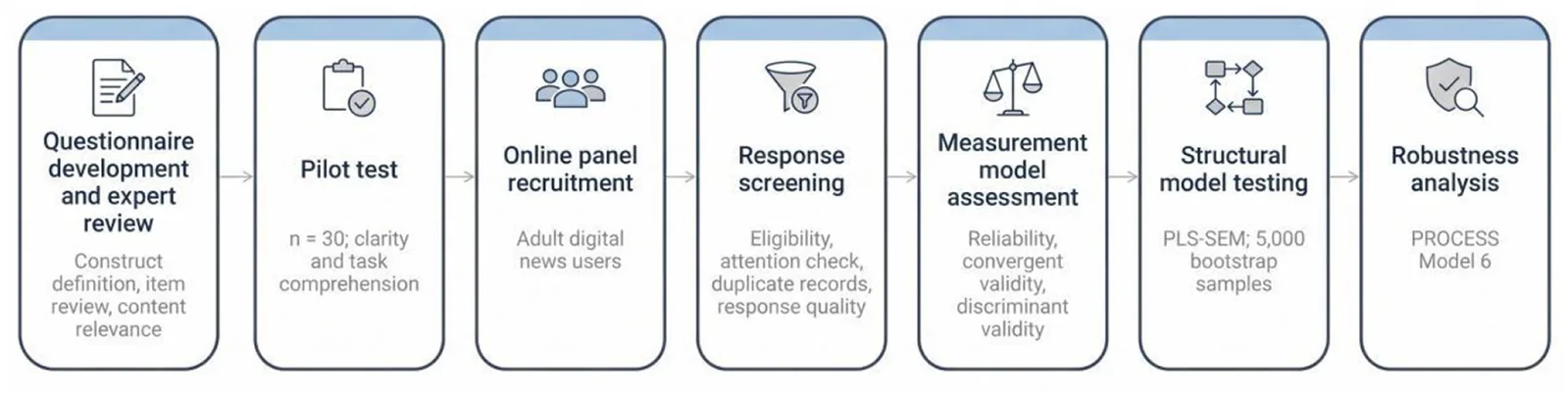
Methodological workflow of the study.

The overall methodological workflow is presented in Figure 1. The workflow begins with questionnaire development and expert review, followed by pilot testing, online panel recruitment, response screening, measurement assessment, structural model testing, and robustness analysis. Figure 1 is included to clarify the sequence of data collection and analysis rather than to present the theoretical model. Finally, we emphasize that the associations modeled in this study are based on cross-sectional survey data. While we utilize sequential mediation models to test theory-driven hypotheses, these associations do not confirm temporal precedence. We interpret all structural paths as statistical associations rather than causal mechanisms, and alternative causal orderings remain theoretically plausible.

### 3.2 Participants and sampling procedure

Data were collected through an anonymous commercial online survey panel of adult digital news users residing in Australia. The sample was recruited via a reputable commercial panel provider that maintains a nationally representative database of Australian adults. The questionnaire was distributed online to respondents who had recently used digital media for news or public-affairs information. Because the analysis used de-identified survey responses and reports only aggregate results, no names, account handles, contact details, or platform identifiers are included in the analytic dataset. Screening questions were placed at the beginning of the questionnaire to confirm eligibility before respondents entered the main survey. Participants were eligible if they met three criteria: they were at least 18 years old, had consumed news through at least one digital platform during the previous month, and were able to complete the questionnaire independently. Respondents who reported no digital news consumption during the previous month were screened out automatically.

A purposive sampling strategy was used because the study required respondents with recent experience of platform-mediated news exposure. Recruitment was distributed across several digital news-use contexts, including social media feeds, news applications, search engines, short-video platforms, personalized recommendation pages, and trending-topic interfaces. This approach was used to avoid a sample composed only of users from one platform type. The panel system also restricted repeated participation by checking account identity, IP address, device information, and completion records.

A total of 468 responses were initially collected. After screening, 48 responses were removed because of incomplete answers, failed attention-check items, duplicate IP or device records, unrealistically short completion time, or straight-line answering across Likert-scale items. The final valid sample consisted of 420 respondents, resulting in a valid response rate of 89.7%.

An a priori power analysis was conducted to ensure the adequacy of the sample size for PLS-SEM estimation. Following the guidelines provided by Hair, the minimum sample size was determined based on the model's most complex structural equation, which contains the largest number of predictors (i.e., five predictors for trust in digital media, including mediators and control variables) (Hair et al., 2022). Adopting a power level of 0.80 and a significance level of α = 0.05 to detect a medium effect size (*f*^2^ = 0.15), the required sample size was estimated at 119 respondents using the inverse square root method for PLS-SEM path coefficients. The retained sample of 420 respondents substantially exceeded this minimum requirement, providing sufficient statistical power for the structural model estimation.

### 3.3 Data collection procedure

The questionnaire included four sections. The first section contained the eligibility screening questions and informed consent statement. Respondents were informed that participation was voluntary, that they could discontinue before submission, that responses would be analyzed anonymously, and that no personally identifying information would be reported. The study used adult survey respondents, did not involve deception or experimental manipulation, and reported results only in aggregate form. The second section measured political identity, algorithmic news exposure, motivated reasoning, misinformation susceptibility, trust in digital media, and control variables. The third section included a headline judgment task designed to assess respondents' ability to distinguish accurate from misleading political or public-affairs news headlines. The fourth section collected demographic information and general media-use characteristics.

Before formal data collection, the questionnaire was reviewed by two digital media researchers and one survey-methods specialist. The review focused on construct coverage, item clarity, response burden, and the distinction between algorithmic news exposure and general digital news use. A pilot test with 30 respondents was then conducted to examine item clarity, questionnaire length, mobile-device display, and comprehension of the headline judgment task. Minor wording changes were made after the pilot test, but no construct was removed. The final questionnaire required approximately 8 to 12 min to complete.

Several procedural controls were applied to improve data quality. Respondents were informed that there were no right or wrong answers for attitudinal items. Scale items were randomized within each construct to reduce order effects. One attention-check item was embedded in the questionnaire. The headline judgment task was placed after the main attitudinal measures to reduce priming effects on political identity and motivated reasoning. The questionnaire platform prevented submission if more than 20% of the main scale items were unanswered.

### 3.4 Measures

All main constructs were measured using multi-item indicators. Unless otherwise stated, items were rated on a seven-point scale. For agreement items, responses ranged from 1 = strongly disagree to 7 = strongly agree. For frequency items, responses ranged from 1 = never to 7 = very frequently. Higher scores indicated higher levels of the corresponding construct.

For constructs that required adaptation to the algorithmically mediated news context, items were developed from the conceptual definitions, reviewed by the expert panel, and refined after pilot testing for clarity, content relevance, and response burden. The operational definitions, item numbers, response formats, example indicators, and scoring rules are summarized in Table 1.

**Table 1:** Operational definitions and measurement structure of the study variables.

Construct	Operational definition	Number of items	Response format	Example indicator	Score interpretation
Political identity	Strength of attachment to a political group, political orientation, or ideological position	4	7-point Likert	“My political views are an important part of who I am.”	Higher score = stronger political identity
Algorithmic news exposure	Frequency of encountering news through personalized feeds, recommendation pages, trending lists, and automated curation	5	7-point frequency scale	“I often encounter news through platform recommendations.”	Higher score = greater algorithmic news exposure
Motivated reasoning	Tendency to evaluate political news in a way that protects prior beliefs	5	7-point Likert	“I am more likely to question news that challenges my political views.”	Higher score = stronger motivated reasoning
Misinformation susceptibility	Subjective and task-based vulnerability to misleading political or public-affairs information online	4 self-report items + 8 headline judgments	7-point Likert and headline accuracy rating	“I sometimes find it difficult to judge whether political news online is true.”	Higher score = greater susceptibility
Trust in digital media	Confidence in the credibility and reliability of digital news environments	5	7-point Likert	“Digital news platforms usually provide reliable information.”	Higher score = higher trust
Political interest	Degree of attention to public affairs and political issues	3	7-point Likert	“I pay attention to political news.”	Higher score = greater political interest
Digital news use frequency	General frequency of consuming news through digital platforms	1	7-point frequency scale	“How often do you consume news through digital platforms in a typical week?”	Higher score = more frequent digital news use
Media literacy	Ability to evaluate source credibility and identify questionable online information	5	7-point Likert	“I check the source before trusting online news.”	Higher score = higher media literacy

#### 3.4.1 Political identity

Political identity was measured as the perceived strength of the respondent's attachment to a political group, political orientation, or ideological position. The measurement was adapted from the expressive partisanship and partisan identity literature, which treats political identity as an affective and social attachment rather than only as a voting preference or ideological label (Huddy et al., 2015). Four items were used: “My political views are an important part of who I am,” “I feel emotionally attached to people who share my political views,” “When my political group is criticized, I feel personally affected,” and “I often interpret public affairs through my political standpoint.” The mean of the four items was used as the political identity score.

#### 3.4.2 Algorithmic news exposure

Algorithmic news exposure was measured as the extent to which respondents encountered news through personalized and platform-curated mechanisms rather than through direct source selection. Five items were developed for this study to capture exposure through personalized feeds, recommendation pages, trending lists, “For You” or similar algorithmic feeds, and automatically suggested news or public-affairs content. Respondents rated how often they encountered news through each mechanism from 1 = never to 7 = very frequently. The mean of the five items was used as the algorithmic news exposure score. This variable was conceptually separated from digital news use frequency, which was included as a control variable measuring general frequency of digital news consumption.

#### 3.4.3 Motivated reasoning

Motivated reasoning was measured as the tendency to evaluate political news in a way that protects prior beliefs or identity-consistent conclusions. Five items assessed selective acceptance, defensive evaluation, and asymmetric scrutiny of political information. The items were: “I am more likely to question news that challenges my political views,” “I tend to trust political news more when it supports my existing beliefs,” “I look for reasons to reject political news that conflicts with my views,” “I feel uncomfortable when reliable news challenges my political standpoint,” and “I usually interpret political news in a way that fits my prior beliefs.” The mean of the five items was used as the motivated reasoning score.

#### 3.4.4 Misinformation susceptibility

Misinformation susceptibility was measured using a combined score derived from self-report items and a headline judgment task. This design was used because misinformation susceptibility may involve both perceived vulnerability and actual difficulty in distinguishing misleading from accurate information. Headline-based misinformation tasks have been used to examine whether users can recognize misleading online claims and respond to accuracy-related interventions (Roozenbeek and van der Linden, 2019).

The self-report component included four items: “I sometimes find it difficult to judge whether political news online is true,” “I have believed online news that later turned out to be misleading,” “I sometimes share or accept political information before checking its source,” and “It is difficult for me to distinguish reliable political news from misleading content online.” These items were rated from 1 = strongly disagree to 7 = strongly agree.

The headline judgment task included eight short headlines on political or public-affairs issues. Four headlines were accurate and four were misleading. The stimulus set was organized by topic area, accuracy label, source category, and adaptation notes so that each item could be checked during questionnaire development. The headlines were adapted from publicly available fact-checking examples and mainstream public-affairs news items. Names, exact dates, specific political party references, and highly identifying details were removed or generalized to reduce prior familiarity and partisan cueing. Crucially, to prevent the discernment score from merely reflecting ideological congruence, the stimuli were explicitly designed to avoid distinct partisan alignment. Consequently, the misleading headlines did not favor conservative or liberal ideologies, ensuring that the task measured structural susceptibility to misinformation rather than partisan evaluation of the stimuli themselves. The eight headlines covered four broad topics: public health, election procedure, economic policy, and public safety. Each topic included one accurate headline and one misleading headline. To reduce confounding from readability and emotional intensity, all headlines were written in a neutral news style and kept within a similar length range of approximately 18 to 25 words.

Two researchers independently classified each headline as accurate or misleading, achieving a high degree of inter-rater reliability (Cohen's κ = 0.88, *p* < 0.001). Disagreements were resolved through deliberation until full consensus was reached. The complete list of headline stimuli and their corresponding classification labels is provided as part of the study materials to ensure transparency and replicability. During the pilot test, headlines that were judged as too obvious, too obscure, or too ambiguous were revised. The misleading headlines were designed to be plausible but inaccurate rather than absurd or easily dismissible.

Respondents rated the perceived accuracy of each headline from 1 = definitely false to 7 = definitely true. Accuracy ratings for misleading headlines were reverse-coded, and a discernment score was calculated by averaging the correctly oriented ratings across the eight headlines. A lower discernment score indicated weaker ability to distinguish accurate from misleading information. For consistency with the structural model, the discernment score was reverse-coded so that higher values represented greater misinformation susceptibility.

The final misinformation susceptibility score was constructed as a formative composite of the self-report score and the reverse-coded headline-task score. While these components represent distinct psychological dimensions—specifically, subjective metacognitive vulnerability (self-report) and objective cognitive performance (headline task)—their integration captures the holistic vulnerability of a user to misinformation in digital environments. We treat this composite as a formative indicator where the two components jointly define the construct, rather than as interchangeable reflections of a single underlying latent trait.

#### 3.4.5 Trust in digital media

Trust in digital media was measured as respondents' confidence in the credibility, reliability, and integrity of digital news sources and platform-mediated news environments. The measure was adapted from the multidimensional news media trust scale, which conceptualizes news trust through perceived topic selection, fact selection, accuracy, and journalistic assessment (Kohring and Matthes, 2007). Five adapted items were used: “Digital news platforms usually provide reliable information,” “I generally trust the news I receive through digital media,” “Digital media can be a credible source of public-affairs information,” “News presented on digital platforms is usually linked to or distributed responsibly,” and “I feel confident using digital media to understand public issues.” The mean of the five items was used as the trust in digital media score.

#### 3.4.6 Control variables

The study controlled for age, gender, education, political interest, digital news use frequency, and media literacy. Age was measured in years. Gender was coded as 1 = male, 2 = female, and 3 = other/prefer not to say. Education was measured as the highest completed education level. Political interest was measured using three items assessing attention to public affairs, interest in political issues, and frequency of following political news. Digital news use frequency was measured by asking respondents how often they consumed news through digital platforms during a typical week. Media literacy was measured using five items assessing source checking, awareness of sponsored or manipulated content, ability to identify credible sources, attention to author and source information, and caution toward unverified online claims. News literacy research supports the inclusion of media literacy as a control because literacy-related knowledge and skepticism may influence how users evaluate online news and misinformation (Vraga and Tully, 2021).

### 3.5 Data screening and missing data treatment

Data screening was conducted before model estimation. First, responses were checked for eligibility, completeness, duplicate submissions, and attention-check performance. Second, completion time was examined. Responses completed in less than one-third of the median completion time were treated as low-quality responses and removed. Third, straight-line responses were identified when a respondent selected the same answer for more than 90% of Likert-scale items. Fourth, univariate outliers were inspected using standardized scores, and values beyond ±3.29 were reviewed for plausibility rather than automatically deleted. Fifth, missing data patterns were examined at both item and respondent levels.

The final dataset retained a small amount of item-level missingness. Cases with more than 20% missing values across the main constructs were removed during screening. For the remaining cases, item-level missing values were handled using expectation-maximization imputation when the missing rate for a construct was below 5%. As a robustness check, the main model was also estimated using mean-composite scores with pairwise deletion. The direction, size, and significance of the core indirect associations were compared across both approaches.

### 3.6 Common method bias

Because the study used survey data collected from the same respondents at a single time point, procedural and statistical remedies were applied to reduce common method bias. Procedurally, the questionnaire separated predictor, mediator, and outcome measures into different sections, randomized item order within constructs, assured respondents of anonymity, and used neutral wording to reduce evaluation apprehension. These remedies follow established recommendations for reducing common method bias in behavioral research (Podsakoff et al., 2003).

Statistically, three checks were performed. First, although Harman's single-factor test is frequently criticized for its limited sensitivity and conservative nature in detecting method bias, we report it here only as a preliminary diagnostic tool, acknowledging that it is not a sufficient diagnostic on its own (Podsakoff et al., 2003). Second, and more critically, full collinearity variance inflation factors (VIFs) were examined for all latent constructs, with VIF values below 3.3 interpreted as robust evidence that common method bias was unlikely to dominate the results (Kock, 2015). Third, a marker-variable sensitivity check was performed by including an unrelated neutral preference item and examining whether the substantive path coefficients changed materially after adjustment.

### 3.7 Data analysis strategy

Data analysis was conducted in five steps. First, descriptive statistics were calculated for all variables, including means, standard deviations, skewness, kurtosis, and bivariate correlations. Second, reliability and convergent validity were assessed using Cronbach's alpha, composite reliability, factor loadings, and average variance extracted. Third, discriminant validity was assessed using both the Fornell-Larcker criterion and the heterotrait-monotrait ratio. Fourth, the structural model was tested by estimating the paths from political identity and algorithmic news exposure to motivated reasoning, from motivated reasoning to misinformation susceptibility, and from misinformation susceptibility to trust in digital media. Fifth, indirect associations were tested using bootstrapping.

Partial least squares structural equation modeling (PLS-SEM) was selected as the primary estimation approach following the criteria for model appropriateness outlined by Hair et al. (2022). First, the research goal is prediction-oriented, aiming to explain the variance in digital media trust via sequential mediation. Second, the model incorporates a mixture of reflective latent constructs and a composite misinformation susceptibility score; the latter, derived from both survey items and headline judgment task performance, is conceptually a formative composite rather than a reflective latent variable. CB-SEM struggles with such mixed-mode models, whereas PLS-SEM offers the flexibility to model these components simultaneously without structural misspecification. Finally, given the non-normality of survey data in complex mediation models, PLS-SEM's distribution-free nature provides a robust estimation path. The model was estimated using SmartPLS 4.0 with 5,000 bootstrap samples, two-tailed significance testing, and bias-corrected confidence intervals. PLS-SEM is suitable for estimating latent-variable models and evaluating measurement and structural components in prediction-oriented social science research (Hair et al., 2022).

Measurement quality was evaluated using established thresholds. Indicator loadings above 0.70 were considered acceptable, although items between 0.60 and 0.70 were retained if deletion did not improve composite reliability or theoretical coverage. Composite reliability values between 0.70 and 0.95 were considered acceptable. Average variance extracted values above 0.50 indicated convergent validity. The Fornell-Larcker criterion was used to compare the square root of AVE with inter-construct correlations (Fornell and Larcker, 1981). HTMT values below 0.85 were treated as evidence of discriminant validity for conceptually related constructs (Henseler et al., 2015). To explicitly address potential conceptual overlap between motivated reasoning and the self-report component of misinformation susceptibility, an exploratory factor analysis (EFA) and an examination of item cross-loadings were conducted as an additional sensitivity check for discriminant validity.

The structural model was assessed using path coefficients, *t* values, *p* values, confidence intervals, coefficient of determination, effect size, and predictive relevance. Multicollinearity was examined using inner variance inflation factor (VIF) values. Mediation was assessed using bootstrapped indirect associations. The sequential indirect association was considered statistically supported when the 95% bootstrap confidence interval did not include zero. To reduce dependence on a single estimation framework, the same sequential mediation structure was re-estimated using PROCESS Model 6 in SPSS (Hayes, 2022). The robustness check used standardized composite scores for political identity, algorithmic news exposure, motivated reasoning, misinformation susceptibility, and trust in digital media so that the direction and approximate size of the indirect associations could be compared with the PLS-SEM estimates. The comparison focused on the direction, size, and confidence intervals of the indirect associations.

To make the screening and analysis procedures reproducible, Table 2 summarizes the main analytic steps, decision rules, and expected outputs.

**Table 2:** Data screening and statistical analysis plan.

Stage	Procedure	Decision rule	Output
Eligibility screening	Remove respondents who did not consume digital news or were under 18	Exclusion before main questionnaire	Eligible respondent pool
Response quality screening	Remove failed attention checks, duplicate submissions, unrealistically short responses, and straight-line answers	Failed attention check; duplicate device/IP; completion time less than one-third median; >90% same response pattern	Valid analytic sample
Missing data check	Inspect item-level and case-level missingness	Remove cases with >20% missing across main constructs; impute item-level missingness below 5%	Cleaned dataset
Reliability assessment	Cronbach's alpha and composite reliability	Values ≥0.70 preferred	Internal consistency evidence
Convergent validity	Factor loadings and AVE	Loading ≥0.70 preferred; AVE ≥0.50	Construct validity evidence
Discriminant validity	Fornell-Larcker and HTMT	Square root of AVE greater than correlations; HTMT < 0.85	Construct distinctiveness
Common method bias	Procedural controls, Harman's test, full collinearity VIF, marker variable	Single factor not dominant; full collinearity VIF < 3.3	CMB diagnostic evidence
Structural model	Estimate path coefficients and indirect associations	Bootstrapped 95% CI not including zero	Hypothesis testing results
Robustness check	PROCESS Model 6 sequential mediation	Direction and confidence intervals compared with PLS-SEM	Robustness evidence

We acknowledge that PLS-SEM, compared to CB-SEM, may produce upwardly biased path coefficients in sequential mediation models (Rönkkö and Evermann, 2013). While our primary interest lies in the robustness of the indirect pathways, we recognize that the reported small-magnitude indirect effects (|β| = 0.034 and |β| = 0.011) should be interpreted with caution. To address this, we conducted an additional CB-SEM sensitivity analysis using the lavaan package in R, treating the misinformation susceptibility composite as a single-indicator observed variable.

### 3.8 Empirical model specification

**Figure 2:**
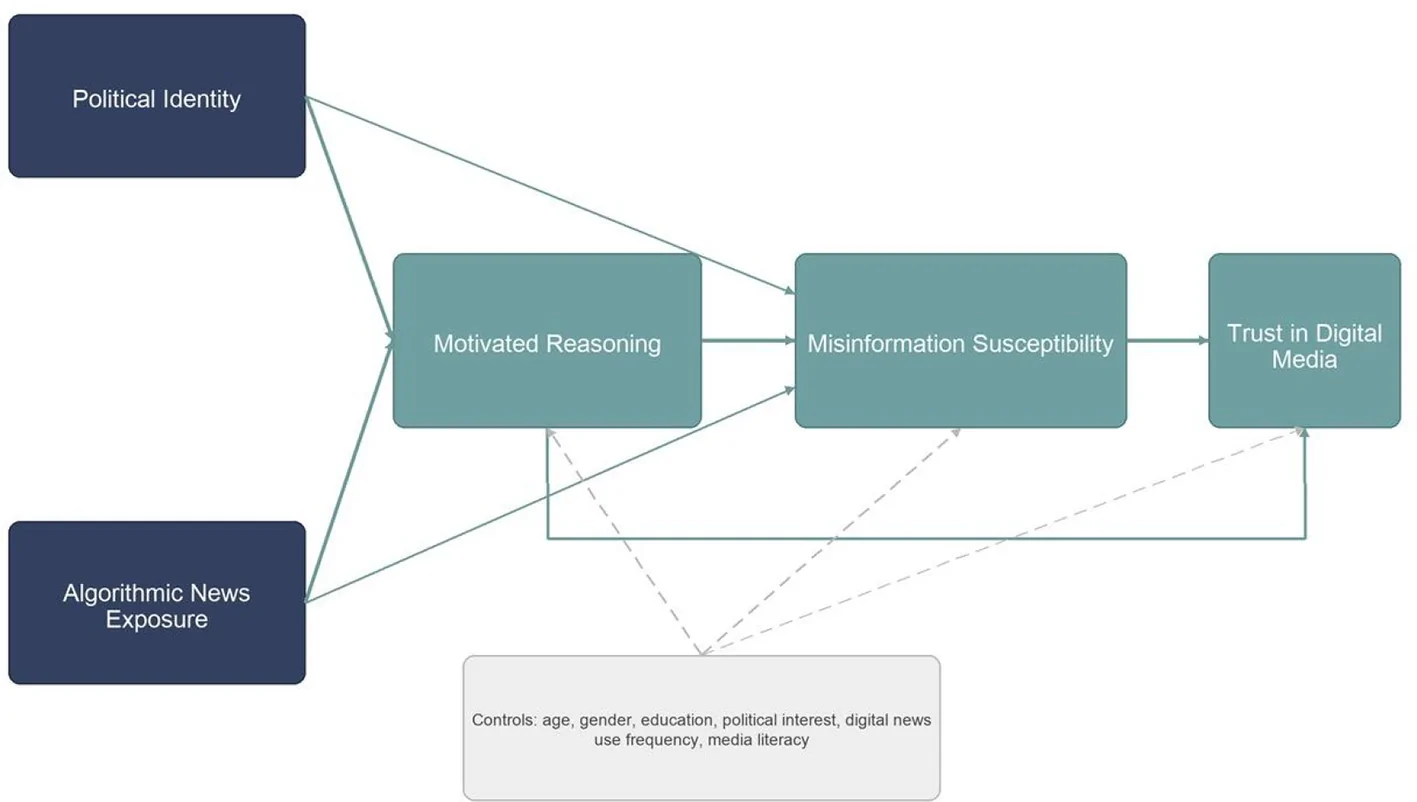
Empirical model linking political identity, algorithmic news exposure, motivated reasoning, misinformation susceptibility, and trust in digital media. Dashed gray arrows indicate control-variable paths. Direct paths from political identity and algorithmic news exposure to trust in digital media were estimated but omitted from the figure for readability.

Figure 2 presents the empirical model tested in this study. Political identity and algorithmic news exposure were specified as exogenous variables. Motivated reasoning and misinformation susceptibility were specified as sequential mediators. Trust in digital media was specified as the endogenous outcome variable. Control variables were linked to motivated reasoning, misinformation susceptibility, and trust in digital media to reduce omitted-variable bias.

The structural equations were specified as follows:


$$\begin{array}{r} \text{MR}={\text{β}}_{0}+\beta_{1}\text{PI}+\beta_{2}\text{ANE}+\beta_{3}\text{Controls}+\text{ε}\\ \text{MS}=\beta_{0}+\beta_{1}\text{MR}+\beta_{2}\text{PI}+\beta_{3}\text{ANE}+\beta_{4}\text{Controls}+\text{ε}\\ \text{TDM}=\beta_{0}+\beta_{1}\text{MS}+\beta_{2}\text{MR}+\beta_{3}\text{PI}+\beta_{4}\text{ANE}+\beta_{5}\text{Controls}+\text{ε} \end{array}$$

where PI refers to political identity, ANE refers to algorithmic news exposure, MR refers to motivated reasoning, MS refers to misinformation susceptibility, and TDM refers to trust in digital media. The sequential indirect association for political identity was specified as PI → MR → MS → TDM. The sequential indirect association for algorithmic news exposure was specified as ANE → MR → MS → TDM. Because the study used a cross-sectional design, the model was interpreted as a theory-informed mediation model of indirect associations rather than as evidence of causal ordering.

## 4 Results

### 4.1 Sample profile and data screening results

A total of 468 responses were initially collected through the online survey panel. After applying the predefined screening criteria, 48 responses were excluded because of incomplete answers, failed attention-check items, duplicate IP or device records, unrealistically short completion time, or straight-line answering across Likert-scale items. The final analytic sample consisted of 420 valid respondents, corresponding to a valid response rate of 89.7%.

The demographic and media-use profile of the valid sample is summarized in Table 3. The respondents ranged in age from 18 to 68 years, with a mean age of 34.59 years. The gender distribution was relatively balanced, with 203 male respondents, 207 female respondents, and 10 respondents selecting other or prefer not to say. The most frequently reported primary news-access channels were social media feeds and news applications, followed by search engines, short-video platforms, and platform recommendation or trending interfaces.

**Table 3:** Sample characteristics of respondents.

Variable	Category/ statistic	*n*/Value	%/SD
Final valid sample	Respondents retained for analysis	420	100.0
Age	Mean	34.59	10.48
Gender	Male	203	48.3
	Female	207	49.3
	Other/prefer not to say	10	2.4
Education	Secondary or below	53	12.6
	College/diploma	122	29.0
	Undergraduate degree	174	41.4
	Postgraduate degree	71	16.9
Primary news platform	Social media feeds	108	25.7
	News applications	112	26.7
	Search engines	81	19.3
	Short-video platforms	75	17.9
	Recommendation/ trending interfaces	44	10.5
Digital news use frequency	Mean	4.68	1.22
Political interest	Mean	4.06	1.44
Media literacy	Mean	4.38	1.12

Item-level missingness was low across the main measurement items. The overall item-level missing rate across the main constructs was 3.43%. No construct had missingness above the 5% threshold after case-level screening. Missing values were therefore handled using expectation-maximization imputation, as specified in the Method section. The main analyses were repeated using mean-composite scores with pairwise deletion, and the direction and significance pattern of the core indirect associations remained consistent.

### 4.2 Descriptive statistics and correlations

Table 4 presents the descriptive statistics and bivariate correlations among the main variables. The mean scores for the five core variables ranged from 3.99 to 4.06 on the seven-point scale, suggesting moderate levels of political identity, algorithmic news exposure, motivated reasoning, misinformation susceptibility, and trust in digital media. Skewness and kurtosis values were within acceptable ranges, indicating no severe deviation from normality at the composite-score level.

**Table 4:** Descriptive statistics and correlations among main variables.

Variable	Mean	SD	1	2	3	4	5
Political identity	4.06	1.37	1.000				
Algorithmic news exposure	3.99	1.32	0.042	1.000			
Motivated reasoning	4.03	1.32	0.444	0.174	1.000		
Misinformation susceptibility	4.05	1.28	0.278	0.236	0.472	1.000	
Trust in digital media	4.04	1.31	−0.294	−0.040	−0.473	−0.467	1.000

Political identity was positively correlated with motivated reasoning (*r* = 0.444) and misinformation susceptibility (*r* = 0.278), and negatively correlated with trust in digital media (*r* = −0.294). Algorithmic news exposure was positively correlated with motivated reasoning (*r* = 0.174) and misinformation susceptibility (*r* = 0.236), although its zero-order correlation with trust in digital media was weak (*r* = −0.040). Motivated reasoning was positively associated with misinformation susceptibility (*r* = 0.472) and negatively associated with trust in digital media (*r* = −0.473). Misinformation susceptibility was also negatively associated with trust in digital media (*r* = −0.467). These patterns were consistent with the proposed direction of the structural model.

### 4.3 Common method bias assessment

Common method bias was assessed using both procedural and statistical checks. The procedural controls were implemented during questionnaire design, including separation of predictor, mediator, and outcome sections, item randomization within constructs, anonymous responses, neutral wording, and inclusion of a headline judgment task. The statistical checks indicated that common method bias was unlikely to dominate the results. Harman's single-factor test showed that the first unrotated factor explained 27.05% of the total variance, which was below the 50% threshold; however, we emphasize that this result should be interpreted with caution given the limitations of this diagnostic. In addition, all full collinearity VIF values ranged from 1.18 to 2.06, below the 3.3 threshold. The marker-variable sensitivity check did not materially change the substantive path estimates. These findings suggested that common method bias was not a major threat to the interpretation of the model.

### 4.4 Measurement model assessment

The measurement model was assessed in two parts because misinformation susceptibility was operationalized as a composite score derived from self-report items and a headline judgment task. First, the reflective multi-item constructs were evaluated for internal consistency reliability, convergent validity, and discriminant validity. Second, the misinformation susceptibility composite score was examined separately in terms of component structure and task reliability.

Table 5 presents the reliability and convergent validity results for the reflective constructs. Cronbach's alpha values ranged from 0.830 to 0.840, exceeding the 0.70 threshold. Composite reliability values ranged from 0.884 to 0.893, indicating satisfactory internal consistency. Average variance extracted values ranged from 0.605 to 0.668, exceeding the 0.50 threshold. Indicator loadings were within acceptable ranges, with all retained items loading above 0.70. For full transparency and to facilitate replication, the individual item-level loadings for each of the four reflective constructs are detailed in Supplementary Table 1.

**Table 5:** Reliability and convergent validity of reflective constructs.

Construct	No. of items	Loading range	Cronbach's alpha	Composite reliability	AVE
Political identity	4	0.791–0.835	0.830	0.889	0.668
Algorithmic news exposure	5	0.748–0.798	0.840	0.887	0.611
Motivated reasoning	5	0.748–0.819	0.834	0.884	0.605
Trust in digital media	5	0.732–0.833	0.837	0.893	0.626

The misinformation susceptibility score was calculated from two standardized components: the four-item self-report susceptibility score and the reverse-coded headline discernment score. The self-report component showed acceptable internal consistency, with Cronbach's alpha = 0.811. The headline judgment task showed acceptable split-half reliability after Spearman-Brown correction, *r* = 0.714. The correlation between the self-report component and the reverse-coded headline-task component was moderate (*r* = 0.382), suggesting that the two components captured related but not identical aspects of misinformation susceptibility. The standardized average of the two components was therefore retained as the misinformation susceptibility score for the structural model.

Discriminant validity among the reflective constructs was assessed using HTMT. Table 6 shows that all HTMT values among the reflective constructs were below 0.85, indicating acceptable discriminant validity. Because misinformation susceptibility was treated as a composite score rather than a reflective latent construct, it was not included in the HTMT matrix. Its associations with other variables were reported in the correlation matrix in Table 4.

**Table 6:** HTMT ratios for reflective constructs.

Construct	PI	ANE	MR	TDM
Political identity	—			
Algorithmic news exposure	0.078	—		
Motivated reasoning	0.538	0.204	—	
Trust in digital media	0.361	0.068	0.558	—

Given the conceptual proximity between motivated reasoning and the self-report items of misinformation susceptibility, a focused discriminant validity assessment was conducted prior to structural model testing. An exploratory factor analysis (Principal Axis Factoring with Promax rotation) was performed specifically on the five motivated reasoning items and the four self-report misinformation susceptibility items. The results yielded a clear two-factor solution (eigenvalues > 1.0) that explained 62.4% of the total variance. All items loaded strongly on their theoretically assigned factors (loadings > 0.65), with no severe cross-loadings observed (all cross-loadings < 0.30). Furthermore, an examination of the item cross-loadings within the PLS-SEM framework confirmed that each indicator's outer loading on its assigned construct was substantially higher than its cross-loadings on the alternative construct. These empirical results confirm that motivated reasoning and self-reported misinformation susceptibility represent distinct psychological phenomena in this dataset, mitigating concerns of tautological measurement.

### 4.5 Structural model results

Before testing the structural paths, multicollinearity was assessed. All inner VIF values ranged from 1.01 to 1.55, indicating that multicollinearity was not a major concern in the structural model.

**Figure 3:**
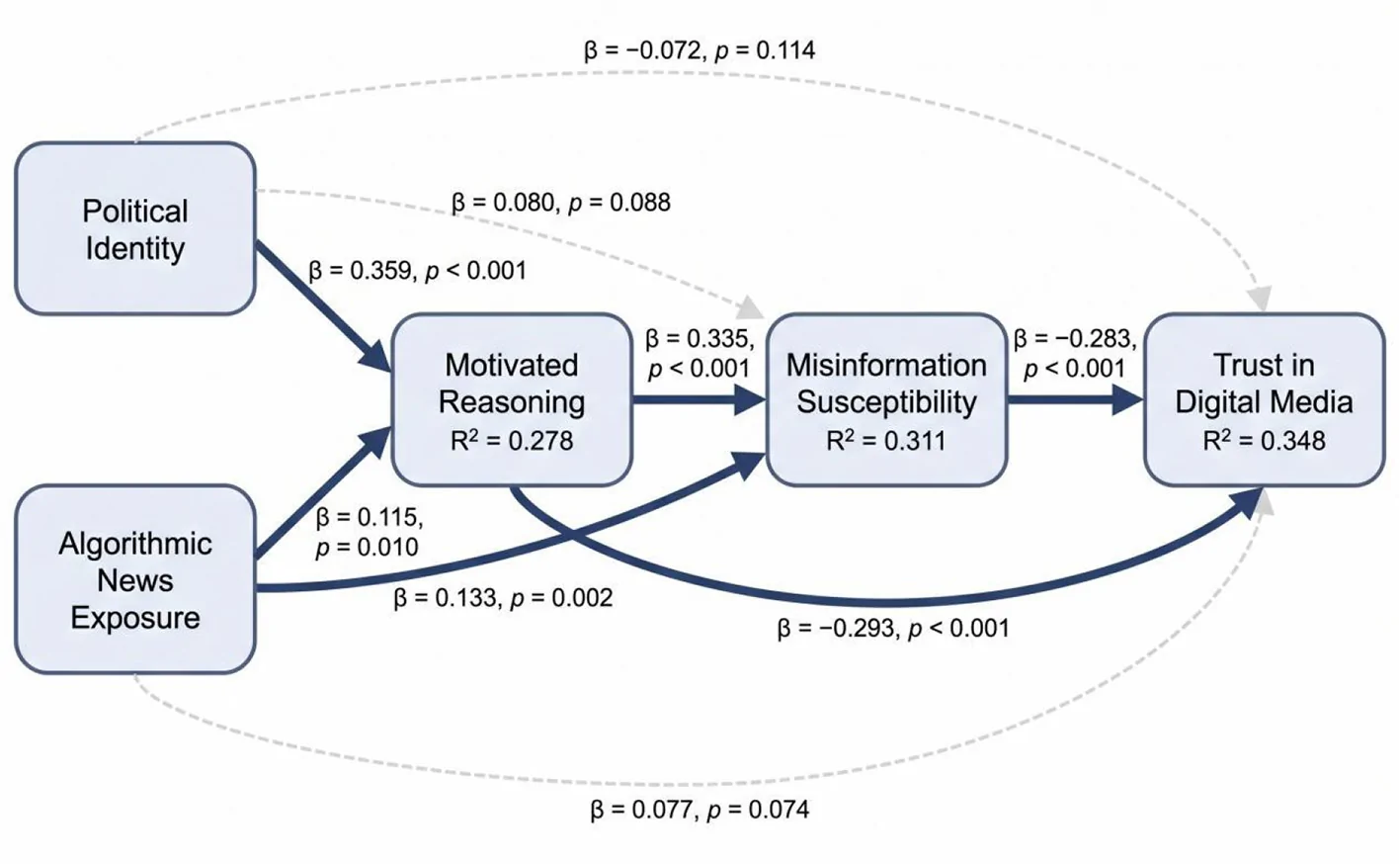
Structural model results with standardized path coefficients. Solid dark arrows indicate statistically supported paths; dashed gray arrows indicate non-supported direct paths.

The structural path results, along with the estimated coefficients for the most theoretically relevant control variables (e.g., media literacy and political interest), are presented in Table 7 and visualized in Figure 3. The full set of control path estimates confirms that the primary structural associations remain robust after accounting for these covariates. Figure 3 is included because the structural model contains multiple direct and indirect paths, and a visual presentation helps show the relative strength and direction of the tested associations. The model explained 27.8% of the variance in motivated reasoning, 31.1% of the variance in misinformation susceptibility, and 34.8% of the variance in trust in digital media.

**Table 7:** Structural model path estimates.

hypothesis/path	β	*t* value	*p* value	95% CI	*f* ^2^	Result
H1: Political identity → Motivated reasoning	0.359	8.07	<0.001	[0.272, 0.447]	0.159	Supported
H2: Algorithmic news exposure → Motivated reasoning	0.115	2.60	0.010	[0.028, 0.202]	0.016	Supported
H3: Motivated reasoning → Misinformation susceptibility	0.335	6.98	<0.001	[0.240, 0.429]	0.117	Supported
Political identity → Misinformation susceptibility	0.080	1.71	0.088	[−0.012, 0.172]	0.007	Not supported
Algorithmic news exposure → Misinformation susceptibility	0.133	3.06	0.002	[0.048, 0.219]	0.023	Supported
H4: Misinformation susceptibility → Trust in digital media	−0.283	5.86	<0.001	[−0.378, −0.188]	0.085	Supported
Motivated reasoning → Trust in digital media	−0.293	5.94	<0.001	[−0.391, −0.196]	0.085	Supported
Political identity → Trust in digital media	−0.072	1.58	0.114	[−0.162, 0.017]	0.006	Not supported
Algorithmic news exposure → Trust in digital media	0.077	1.79	0.074	[−0.007, 0.161]	0.008	Not supported
Age → Trust in digital media	0.042	1.15	0.250	-	-	-
Political interest → Motivated reasoning	0.125	2.45	0.014	-	-	-
Media literacy → Misinformation susceptibility	−0.156	3.12	0.002	-	-	-
Gender → Trust in digital media	0.035	0.82	0.412	-	-	-
Education → Misinformation susceptibility	−0.118	2.64	0.008	-	-	-
Digital news use frequency → Trust in digital media	0.062	1.41	0.159	-	-	-

Political identity was positively associated with motivated reasoning (β = 0.359, *p* < 0.001), supporting H1. Algorithmic news exposure was also positively associated with motivated reasoning (β = 0.115, *p* = 0.010), supporting H2, although the effect size was small. Motivated reasoning was positively associated with misinformation susceptibility (β = 0.335, *p* < 0.001), supporting H3. Misinformation susceptibility was negatively associated with trust in digital media (β = −0.283, *p* < 0.001), supporting H4. The direct path from political identity to misinformation susceptibility was not statistically supported after motivated reasoning and control variables were included. The direct paths from political identity and algorithmic news exposure to trust in digital media were also not statistically supported.

### 4.6 Mediation analysis

**Figure 4:**
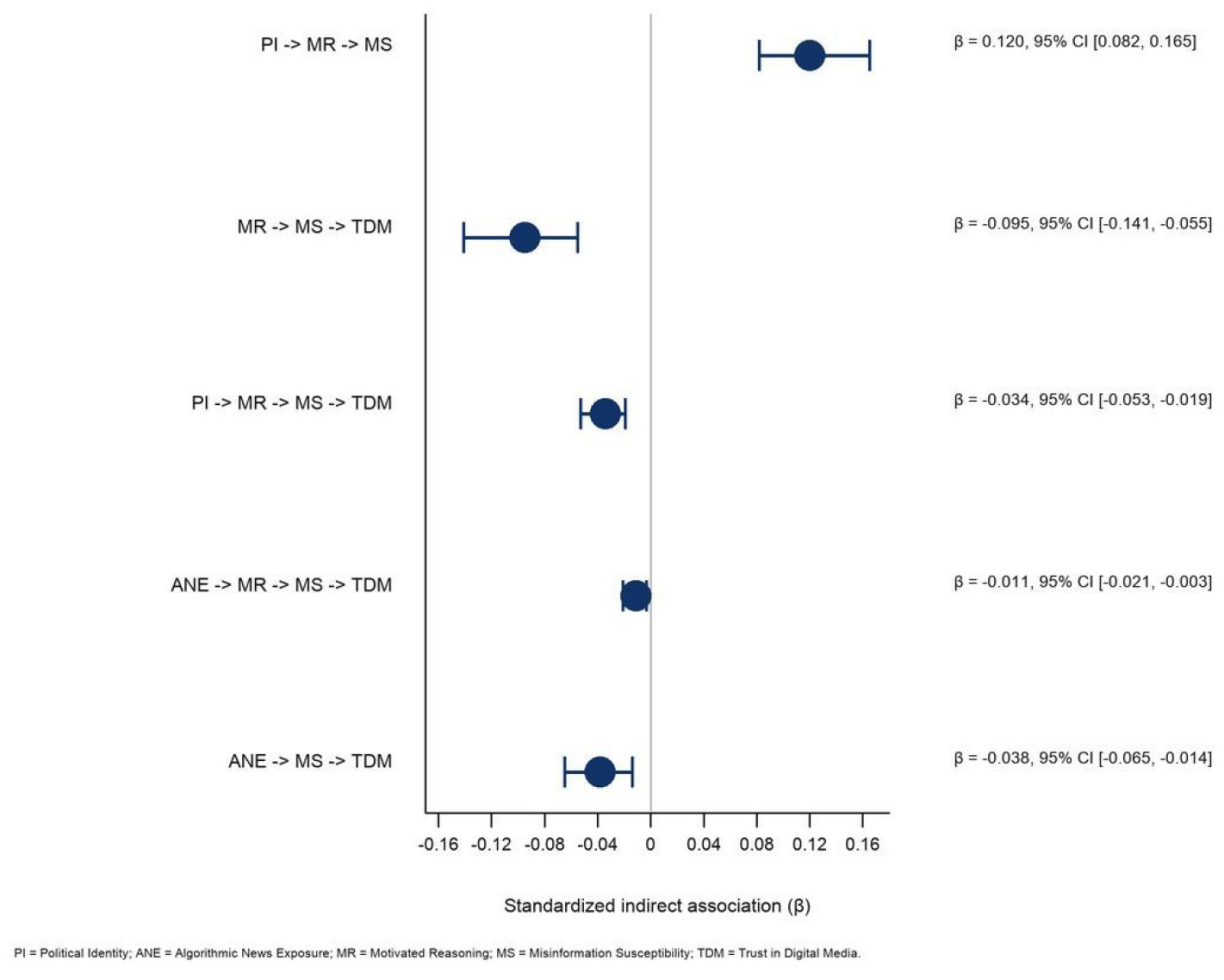
Bootstrapped indirect associations with 95% confidence intervals. PI, political identity; ANE, algorithmic news exposure; MR, motivated reasoning; MS, misinformation susceptibility; TDM, trust in digital media.

The mediation results are summarized in Table 8. Bootstrapping with 5,000 resamples was used to test the indirect associations. To complement the tabular presentation, Figure 4 displays the bootstrapped indirect associations and their 95% confidence intervals. Figure 4 is included because the mediation paths are central to the research model and because the confidence intervals provide a direct visual summary of which indirect associations were statistically supported.

**Table 8:** Bootstrapped indirect associations.

Indirect association path	β Coefficient	95% bootstrap CI	Interpretation
Political identity → motivated reasoning → misinformation susceptibility	0.120	[0.082, 0.165]	Supported
Motivated reasoning → misinformation susceptibility → trust in digital media	−0.095	[−0.141, −0.055]	Supported
Political identity → motivated reasoning → misinformation susceptibility → trust in digital media	−0.034	[−0.053, −0.019]	Supported
Algorithmic news exposure → motivated reasoning → misinformation susceptibility → trust in digital media	−0.011	[−0.021, −0.003]	Supported
Political identity → misinformation susceptibility → trust in digital media	−0.023	[−0.052, 0.003]	Not supported
Algorithmic news exposure → misinformation susceptibility → trust in digital media	−0.038	[−0.065, −0.014]	Supported
Political identity → motivated reasoning → trust in digital media	−0.105	[−0.152, −0.065]	Supported
Algorithmic news exposure → motivated reasoning → trust in digital media	−0.034	[−0.065, −0.008]	Supported

The sequential indirect association from political identity to trust in digital media through motivated reasoning and misinformation susceptibility was statistically supported, β = −0.034, 95% CI [−0.053,−0.019], supporting H7a. The sequential indirect association from algorithmic news exposure to trust in digital media through the same mediators was also supported, β = −0.011, 95% CI [−0.021, −0.003], supporting H7b. The indirect association from political identity to misinformation susceptibility through motivated reasoning was positive and statistically supported, β = 0.120, 95% CI [0.082, 0.165], supporting H5. The indirect association from motivated reasoning to trust in digital media through misinformation susceptibility was negative and statistically supported, β = −0.095, 95% CI [−0.141, −0.055], supporting H6.

### 4.7 Robustness check using PROCESS Model 6

To ensure the robustness of the structural model findings, we employed two distinct analytical frameworks.

#### 4.7.1 CB-SEM sensitivity analysis

As an additional robustness check, a CB-SEM estimation was performed using the lavaan package in R. The sequential indirect paths (PI → MR → MS → TDM and ANE → MR → MS → TDM) were estimated using the maximum likelihood estimator, with the misinformation susceptibility composite treated as a single-indicator observed variable. The analysis confirmed the convergence of results between PLS-SEM and CB-SEM. The global model exhibited satisfactory fit to the data (χ^2^ = 415.22, df = 234, *p* < 0.001, CFI = 0.962, TLI = 0.955, RMSEA = 0.043, SRMR = 0.048). Specifically, the CB-SEM estimation corroborated the sequential indirect path from political identity to trust in digital media (β = −0.031, SE = 0.008, 95% CI [−0.047, −0.015], *p* < 0.001), which aligns closely with the PLS-SEM estimate (β = −0.034). Similarly, the sequential indirect path from algorithmic news exposure to trust remained statistically supported in the CB-SEM framework (β = −0.009, SE = 0.004, 95% CI [−0.017,−0.001], *p* = 0.022), mirroring the PLS-SEM result (β = −0.011). This quantitative consistency across disparate analytical frameworks substantially strengthens confidence in our findings, indicating that the reported sequential mediation is not an artifact of PLS-SEM estimation bias.

#### 4.7.2 Sequential mediation check via PROCESS Model 6

To further assess whether the mediation findings depended on the PLS-SEM estimation framework, the sequential mediation structure was re-estimated using PROCESS Model 6 in SPSS. The robustness check used the same control variables as the main analysis: age, gender, education, political interest, digital news use frequency, and media literacy. The results were consistent with the PLS-SEM estimates.

For political identity, the sequential indirect association through motivated reasoning and misinformation susceptibility remained statistically supported, β = −0.031, 95% CI [−0.050, −0.016]. For algorithmic news exposure, the sequential indirect association through the same mediators also remained supported, β = −0.010, 95% CI [−0.020, −0.002]. The direction and confidence intervals of the PROCESS estimates were consistent with the main PLS-SEM results. This suggested that the mediation pattern was not dependent on a single estimation approach.

#### 4.7.3 Sensitivity analysis of misinformation susceptibility components

Given the conceptual distinction between subjective and objective susceptibility, we conducted a sensitivity analysis by re-estimating the structural model with the self-report vulnerability score and the objective headline discernment score treated as separate endogenous mediators. This analysis confirmed that the direction and significance of the sequential indirect paths remained consistent with our main findings, regardless of whether the two components were integrated into a composite or analyzed separately. This convergence suggests that the sequential association between identity-based processing and digital media trust is robust and not driven by the specific operationalization of misinformation susceptibility.

### 4.8 Summary of hypothesis testing

The hypothesis testing results are summarized in Table 9. H1, H2, H3, H4, H5, H6, H7a, and H7b were supported. The strongest structural associations were observed for political identity → motivated reasoning, motivated reasoning → misinformation susceptibility, motivated reasoning → trust in digital media, and misinformation susceptibility → trust in digital media. Algorithmic news exposure showed weaker but statistically supported associations with motivated reasoning and misinformation susceptibility. Overall, the results supported the proposed indirect-association model in which motivated reasoning and misinformation susceptibility linked political identity and algorithmic news exposure to trust in digital media.

**Table 9:** Summary of hypothesis testing.

Hypothesis	Statement	Result
H1	Political identity is positively associated with motivated reasoning.	Supported
H2	Algorithmic news exposure is positively associated with motivated reasoning.	Supported
H3	Motivated reasoning is positively associated with misinformation susceptibility.	Supported
H4	Misinformation susceptibility is negatively associated with trust in digital media.	Supported
H5	Motivated reasoning mediates the relationship between political identity and misinformation susceptibility.	Supported
H6	Misinformation susceptibility mediates the relationship between motivated reasoning and trust in digital media.	Supported
H7a	Motivated reasoning and misinformation susceptibility sequentially mediate the relationship between political identity and trust in digital media.	Supported
H7b	Motivated reasoning and misinformation susceptibility sequentially mediate the relationship between algorithmic news exposure and trust in digital media.	Supported

## 5 Discussion

This study examined how political identity and algorithmic news exposure were associated with trust in digital media through motivated reasoning and misinformation susceptibility. The results supported the proposed indirect-association model. Political identity showed a strong positive association with motivated reasoning, while algorithmic news exposure showed a weaker but still statistically supported association with motivated reasoning. Motivated reasoning was positively associated with misinformation susceptibility, and both motivated reasoning and misinformation susceptibility were negatively associated with trust in digital media. The direct associations from political identity and algorithmic news exposure to trust in digital media were not statistically supported after the mediators and controls were included. This pattern suggests that trust in digital media is better understood through the psychological and informational processes by which users interpret political news rather than through direct associations with identity or platform exposure alone.

The first important finding concerns the role of political identity. Political identity was strongly associated with motivated reasoning, which indicates that identity-based attachment may relate to how users evaluate political news. While this association appears consistent across our sample, it remains to be seen whether this relationship holds symmetrically across diverse ideological orientations, as recent work suggests that the psychological mechanisms of motivated reasoning may vary between left- and right-leaning groups. This finding is consistent with broader research showing that fake news and misinformation cannot be treated only as content-level problems, because user interpretation, belief formation, and social context also matter in the online news ecosystem (Lazer et al., 2018). In this study, political identity was not directly associated with trust in digital media after motivated reasoning and misinformation susceptibility were included. This result suggests that political identity may be linked to trust primarily through the way users process political information. In practical terms, identity does not necessarily lower or raise trust by itself. Rather, its relevance appears when political identity becomes part of the reasoning process through which users judge information. It is important to address the conceptual complexity of our motivated reasoning scale. The items utilized herein capture a multifaceted construct, reflecting both cognitive biases and affective states. As suggested by recent critical feedback, some items may conflate identity-protective tendencies with general political skepticism or epistemic scrutiny. Furthermore, the inclusion of items assessing “discomfort” acknowledges that identity-protective cognition is frequently intertwined with affective reactions. While these facets (cognition and affect) are distinct in laboratory-based paradigms, they are arguably inseparable in naturalistic digital news environments where political information is deeply personalized. We acknowledge this as a boundary condition of our study: our scale measures a broad identity-processing tendency rather than a narrowly isolated cognitive operation. Importantly, because the present study did not include specific comparison instruments for general political skepticism or media cynicism, the empirical distinctiveness of our motivated reasoning scale from these related constructs could not be directly tested. Although our exploratory factor analyses confirmed the conceptual separation between motivated reasoning and self-reported misinformation susceptibility, this does not fully resolve the potential measurement overlap with broader media cynicism. Therefore, future research must explicitly incorporate validated scales for political skepticism and media cynicism to rigorously establish discriminant validity and disentangle these cognitive, affective, and skeptical components.

It is noteworthy that the zero-order correlation between political identity and algorithmic news exposure was negligible (r = 0.042). Theoretically, this null correlation provides a crucial insight: algorithmic news exposure is not merely an extension or byproduct of political identity. Instead, these variables appear to function as distinct antecedents within the digital news environment. While prior literature often assumes that partisan users are ‘driven' into algorithmic echo chambers, our findings suggest that algorithmic news encounters are widespread across the ideological spectrum, independent of the strength of one's political identity. Consequently, our model's significance lies in showing that both identity-based reasoning and algorithmically structured exposure independently contribute to the motivated processing of news, rather than one being a prerequisite for the other.

The second finding concerns algorithmic news exposure. Algorithmic news exposure was positively associated with motivated reasoning, but the size of this association was smaller than that of political identity. This is an important result because it avoids an overly deterministic interpretation of algorithms. Prior work on Facebook news exposure has shown that platform algorithms, social networks, and user choices jointly shape exposure to ideologically diverse information (Bakshy et al., 2015). The present finding fits this more cautious view. Algorithmic exposure may create conditions in which identity-consistent interpretation becomes more likely, but it should not be treated as a stand-alone force that automatically determines trust. Furthermore, while behavioral logs offer precision, the subjective perception of algorithmic exposure remains a vital psychological construct; it is how users perceive their information environment—rather than the raw data of what they encounter—that often drives their cognitive and attitudinal responses. Users' own political attachments, prior beliefs, media habits, and platform interactions remain part of the same information environment.

The positive yet non-significant path from algorithmic news exposure to trust (β= 0.077, *p* = 0.074) further complicates the deterministic view of algorithms. While our model hypothesized a negative association via misinformation susceptibility, the direct path coefficient suggests that, for some users, algorithmic curation might be perceived as a source of convenience or utility, which could—at least in direct terms—partially offset the corrosive effects of misinformation susceptibility. This non-significant direct path underscores that the “algorithmic effect” is not uniformly detrimental to trust; rather, it is contingent upon how individuals filter these algorithmic inputs through their cognitive and identity-driven reasoning processes. Online news consumption studies have shown that search engines and social media may increase exposure to diverse sources while also contributing to ideological segregation among some users (Flaxman et al., 2016). The present results are consistent with this mixed pattern. Algorithmic exposure may provide access to more news, but repeated exposure to personally relevant or politically congenial content may also create conditions for selective interpretation. Its effect is therefore better captured through mediated pathways than through a simple direct association with trust. This result aligns with recent empirical work in health behavior research, which demonstrates that trust in social media information is fundamentally structured by baseline demographic differences and active psychosocial evaluations—such as users' support for platform misinformation restrictions—rather than by simple exposure metrics alone (Chung and Barry, 2026). As these recent findings indicate, trust in digital environments is increasingly determined by individual-level cognitive appraisals and demographic characteristics, reinforcing our premise that media trust relies on active psychological processing rather than the mere passive frequency of algorithmic curation. This reinforces our finding that the “algorithm effect” is indirect, manifesting through how users process content rather than through the technical environment of exposure itself.

The third finding concerns misinformation susceptibility. The results showed that motivated reasoning was positively associated with misinformation susceptibility, and misinformation susceptibility was negatively associated with trust in digital media. This supports the idea that the problem is not only whether users encounter misleading information, but also whether they are able to evaluate it under identity-relevant conditions. Although motivated reasoning and self-reported susceptibility share certain conceptual similarities, our auxiliary measurement checks confirmed their empirical distinctiveness. This indicates that their positive association (β = 0.335) reflects a substantive psychological sequence rather than an artifact of measurement overlap. This supports the idea that the problem is not only whether users encounter misleading information, but also whether they are able to evaluate it under identity-relevant conditions. Large-scale evidence on online news diffusion shows that false news can spread farther, faster, and more broadly than true news, partly because of the novelty and emotional reactions it evokes (Vosoughi et al., 2018). In this study, misinformation susceptibility was linked to lower trust in digital media, suggesting that users who have greater difficulty distinguishing misleading from accurate information may also report lower confidence in digital news environments.

A notable finding in our model is the significant direct association between motivated reasoning and trust in digital media (β = −0.293), which operates independently of misinformation susceptibility. This suggests that motivated reasoning does more than simply increase vulnerability to specific misleading claims; it may have a broader negative association with media trust beyond its association through misinformation susceptibility. From an identity-protective perspective, the constant act of filtering news through a partisan lens may cultivate a pervasive sense of media skepticism. Even when users are not actively encountering misinformation, the habitual process of engaging in identity-defensive evaluation—questioning challenging views and prioritizing congenial content—may diminish their general confidence in the digital media landscape. This indicates that motivated reasoning functions as both an indirect association with trust, via misinformation, and an association with media skepticism, likely through a global, identity-protective appraisal of the digital news ecosystem.

Furthermore, our structural model reveals a significant direct association between algorithmic news exposure and misinformation susceptibility (β= 0.133), bypassing motivated reasoning. This finding suggests that algorithmic news environments may foster misinformation vulnerability through mechanisms beyond identity-based cognition. A potential theoretical explanation lies in the structural characteristics of platform ecosystems, which often prioritize high-volume content delivery and incentivize rapid engagement. Such environments may facilitate a ‘low-elaboration' news processing mode, where users are perpetually exposed to a stream of information—including misinformation-adjacent content—that circumvents systematic cognitive verification. Unlike motivated reasoning, which is driven by identity protection, this direct path likely reflects the environmental effect of platform architecture, where the mere frequency and presentation style of algorithmically curated news may normalize questionable content, thereby increasing susceptibility regardless of a user's prior political motivations.

The mediation results further clarify this mechanism. Political identity was linked to trust in digital media through motivated reasoning and misinformation susceptibility. Algorithmic news exposure also showed a smaller but statistically supported sequential indirect association through the same two mediators. These findings indicate that trust-related outcomes are not only associated with what users are exposed to, but also with how they process and evaluate information. This is relevant because corrected misinformation can continue to influence political attitudes even after it has been discredited (Thorson, 2016). Once misleading information is processed through identity-consistent reasoning, its influence may extend beyond a single belief judgment and become part of a broader evaluation of the digital media environment.

The findings also contribute to the literature on misinformation correction and media trust. Research on misinformation correction has shown that false information may continue to affect reasoning even after corrections are provided (Lewandowsky et al., 2012). More recent work has also cautioned that the durability of political misperceptions cannot be explained only by a simple backfire effect (Nyhan, 2021). The present study extends this line of work by showing that misinformation susceptibility is not merely a downstream error in judgment; it is part of a broader trust-related pathway. When users process political information through motivated reasoning and become more vulnerable to misleading content, trust in digital media may become less stable. This interpretation is consistent with the non-significant direct paths in the model, which suggest that the cognitive pathway is more informative than direct identity-to-trust or exposure-to-trust associations.

The study has several theoretical implications. First, it connects two often separated explanations of digital media trust: identity-based cognition and algorithmically structured exposure. Political identity represents a user-level psychological orientation, while algorithmic news exposure represents a platform-mediated condition of information access. By placing both in the same model, the study shows that digital media trust should be examined in relation to both user cognition and platform-shaped exposure. Second, the findings clarify the role of motivated reasoning as a bridge between political identity and misinformation susceptibility. Motivated reasoning is not only an attitudinal bias; in this model, it functions as a mechanism that helps explain why politically meaningful information may become more vulnerable to misleading interpretation. Third, the study contributes to measurement practice by combining self-reported misinformation susceptibility with a headline judgment task, rather than relying only on subjective perceptions of vulnerability.

The study also has practical implications. For digital platforms, the findings suggest that interventions should not focus only on removing false content after it appears. Platform design choices that affect repeated exposure, recommendation transparency, and the visibility of source cues may also matter. Social media correction research has shown that related-story functions and corrective information can help reduce misinformation under certain conditions (Bode and Vraga, 2015). However, the present findings suggest that such interventions may be more effective when they address both content accuracy and users' reasoning context. For news organizations and media literacy educators, the results indicate that training should go beyond general advice such as “check the source.” It should also help users recognize when political identity or prior beliefs are shaping their evaluation of news. Experimental evidence indicates that warnings and fact-check labels can reduce belief in false headlines, although the size of these effects is often modest (Clayton et al., 2020). This means that trust-building strategies should combine source transparency, accuracy prompts, headline discernment training, and platform-level friction before sharing or endorsing political content.

Several limitations should be acknowledged. First, the study used a cross-sectional design. Although the model was theoretically ordered based on existing literature, the cross-sectional data cannot establish temporal precedence or causal sequencing among the variables. As is common with observational data, alternative directional pathways are equally plausible; for instance, lower trust in digital media might drive users into algorithmic echo chambers or heighten protective motivated reasoning as a defensive cognitive mechanism. Future studies should use longitudinal designs, panel surveys, or experiments to rigorously test the temporal stability and the exact causal direction of these associations. Second, the sample was collected through an online survey panel. Although screening and quality-control procedures were applied, panel respondents may differ from the broader population of digital news users. Future research could use probability-based samples or compare results across countries and platform ecosystems. Third, algorithmic news exposure was operationalized via a seven-point self-report frequency scale. While this approach captures subjective perceptions, a critical limitation in current media psychology is that users often struggle to distinguish between algorithmically curated feeds, chronologically ordered timelines, and socially influenced content. Relying on such subjective assessments—rather than objective behavioral trace data or browser logs—may introduce measurement error that potentially confounds the estimated strength of platform exposure pathways. Specifically, if respondents misidentify their exposure patterns, the reported coefficients might underrepresent the true impact of algorithmic curation on motivated reasoning. Future research should strive to triangulate survey measures with validated behavioral metrics or platform-specific data to provide a more granular understanding of how distinct algorithmic architectures interact with cognitive processing. Fourth, the headline judgment task used a limited set of eight political and public-affairs headlines. Although this design improved measurement beyond self-report alone, future research should use larger headline pools, topic-specific misinformation sets, and repeated task designs to improve measurement precision. Additionally, future studies could incorporate symmetrically balanced partisan headlines (i.e., an equal number of conservative-leaning and liberal-leaning false claims) while simultaneously accounting for the ideological distribution of the sample. This would further disentangle structural susceptibility from ideological congruence in varied political contexts. Fifth, the findings are specific to the Australian political and media context. Political identity, algorithmic news ecosystems, and media trust are deeply embedded in national institutional settings; thus, the generalizability of our results to other countries with different party systems or media environments remains to be established. For instance, the political identity scale items may carry different affective weights in multi-party vs. two-party systems, potentially affecting the cross-national measurement equivalence. Future research should replicate this model in diverse international contexts to ascertain whether the sequential mediation pathways observed here are universal or contingent upon the specific structure of the Australian political communication system.

Finally, our measure of political identity captures the strength of partisan attachment rather than its ideological direction (e.g., left-wing vs. right-wing). Recent research suggests that the relationship between partisan identity and motivated reasoning may be asymmetric across the ideological spectrum (Jost et al., 2022; Van Bavel and Pereira, 2018). By treating identity strength as a homogeneous construct, we implicitly assume that strong left- and right-wing identities produce equivalent effects on motivated reasoning—an assumption that remains theoretically contestable. Future research should investigate whether these sequential mediation pathways differ in magnitude or directionality between ideological groups, potentially revealing that the link between identity and information processing is more contingent upon specific political worldviews than current models suggest.

Despite these limitations, the study provides a coherent account of how political identity and algorithmic news exposure are associated with trust in digital media through motivated reasoning and misinformation susceptibility. The results show that trust in digital media is not simply a matter of whether users consume more or less digital news. It is also associated with whether users process political information through identity-protective reasoning - a process that was negatively associated with trust in our model - and whether they remain able to distinguish misleading from accurate content. This finding is especially relevant in algorithmically mediated news environments, where exposure, engagement, identity, and credibility judgments are increasingly intertwined.

## 6 Conclusion

This study investigated the associations among political identity, algorithmic news exposure, motivated reasoning, misinformation susceptibility, and trust in digital media. The findings supported a sequential indirect-association model. Political identity and algorithmic news exposure were associated with motivated reasoning, motivated reasoning was associated with greater misinformation susceptibility, and misinformation susceptibility was associated with lower trust in digital media. The direct associations from political identity and algorithmic news exposure to trust in digital media were not statistically supported after the mediators and control variables were included, suggesting that the cognitive and informational pathway was central to the model.

The study contributes to digital media and political communication research by showing that trust in digital media should be understood through the interaction between identity-based reasoning and platform-mediated news exposure. Political identity provides an interpretive frame, algorithmic exposure structures the conditions of news encounter, motivated reasoning characterizes the evaluation process, and misinformation susceptibility bridges this process to trust-related outcomes. These findings indicate that efforts to improve digital media trust should address not only misinformation content but also the reasoning conditions under which users judge political information.

Future research should test this model with longitudinal, experimental, and platform-specific data. Further work should also examine whether the same pathway operates differently across political contexts, media systems, age groups, and platform types. As digital platforms continue to shape how citizens encounter political news, understanding the cognitive and platform-related conditions of trust will remain important for both scholarship and practice.

## Data Availability

The raw data supporting the conclusions of this article will be made available by the authors, without undue reservation.
